# Multisystemic Inflammatory Syndrome and Thrombotic Microangiopathy as Complications of COVID-19 in a Child: A Case Report

**DOI:** 10.3389/fped.2021.659069

**Published:** 2021-06-04

**Authors:** Samira Shizuko Parreão Oi, Monique Pereira Rêgo Muniz, Igor Murad Faria, Natalino Salgado Filho, Dyego José Araújo de Brito, Joyce Santos Lages, Letícia Pádua Lauande, Thina Klicia Mendonça Oliveira, Kaile de Araújo Cunha, Precil Diego Miranda de Menezes Neves, Gyl Eanes Barros Silva

**Affiliations:** ^1^University Hospital, Federal University of Maranhão (HU-UFMA), São Luís, Brazil; ^2^Nephrology Division, Medical School, University of São Paulo (FM-USP), São Paulo, Brazil

**Keywords:** COVID-19, lupus nephritis, thrombotic microangiopathy, multisystemic inflammatory syndrome, acute kidney injury

## Abstract

Clinical presentations of the novel coronavirus (SARS-CoV-2) infection are quite varied, ranging from asymptomatic conditions to potentially fatal disease. The kidney is one of the affected targets of coronavirus disease (COVID-19) complications, and renal dysfunction is a significant prognostic factor for mortality. This report describes a series of clinical complications in a previously healthy child who developed nephritic syndrome with a concomitant SARS-CoV-2 infection. These complications include acute kidney injury that progressed to chronicity, multisystemic inflammatory syndrome, Kawasaki-like syndrome, and thrombotic microangiopathy.

## Introduction

Clinical presentations of the novel coronavirus (SARS-CoV-2) infection are quite wide, ranging from asymptomatic conditions to potentially fatal disease. In the most severe cases, the exacerbated immune response is believed to generate a massive proliferation of defense cells and cytokines that ultimately affect several organs and systems. The kidney is one of the affected targets of coronavirus disease (COVID-19) complications, and renal dysfunction is a significant prognostic factor for mortality (1, 2).

The current literature describes multiple mechanisms leading to kidney injury in COVID-19 patients, including ischemic injury, a “cytokine storm,” and direct SARS-CoV-2 injury (3). There are reports of viral inclusions in the renal parenchyma but the kidney has not been definitively defined as a direct target of this disease (1, 3, 4).

Here, we report an unusual case of a child who presented with multisystemic inflammatory syndrome associated with thrombotic microangiopathy after SARS-CoV-2 infection.

## Case Report

A previously healthy 7-year-old female child presented with skin lesions, suggestive of impetigo, in her lower limbs and buttocks, which progressed after 2 weeks with asthenia, loss of appetite, generalized edema, macroscopic hematuria, and oliguria. At admission, her anthropometric evaluation revealed weight: 22 Kg (z-score: 1), height: 122 cm (z-score: 1) and her blood pressure was 120/90 mmHg (stage 2 hypertension). At this time, the patient was hospitalized in her home city and diagnosed with an acute post-infectious glomerulonephritis. Laboratory findings that corroborated the diagnostic were a serum creatinine 3.5 mg/dL, anti-streptolysin O 1010.4 IU/mL, anti-deoxyribonuclease B 711 U/mL, normal complement, nephrotic proteinuria (3,864 mg/24 H−138 mg/kg/24 H), urine analysis with hematuria (60 red blood cells per field) and leukocyturia (26 white blood cells per field), positive antinuclear antibody 1:160 with a fine speckled pattern and reagent nucleus, and negative hepatitis B, C, and HIV serology. At that time, there were no reports of patient contact with other persons positive for SARS-CoV-2 infection.

**Figure 1:**
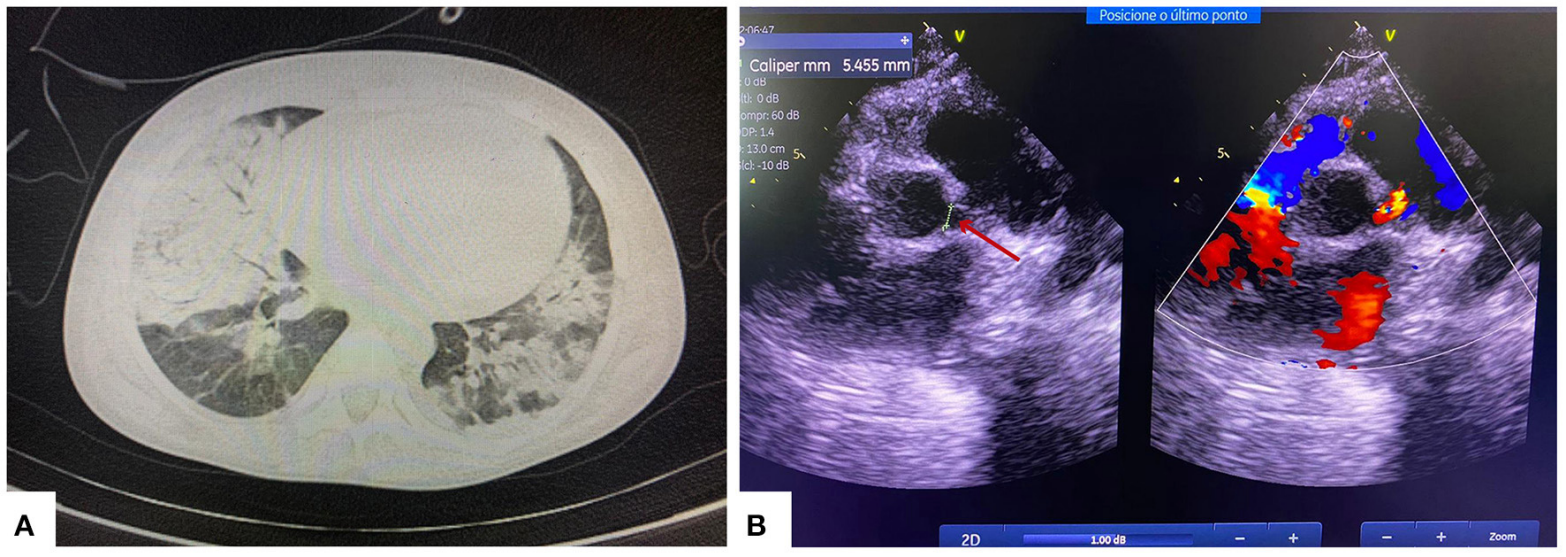
**(A)** Chest computed tomography showing multifocal and confluent consolidations with ground-glass attenuation in >50% of the pulmonary parenchyma. **(B)** Echocardiogram showing left coronary artery dilatation (red arrow).

During her hospital stay, the patient was evaluated by a nephrologist, who conducted the case as a post-infectious glomerulonephritis. She received supportive treatment that included diuretics, anti-hypertensive drugs, low sodium diet and water restriction, even in the setting of laboratory tests showing a progressive worsening of renal function. The patient undergone clinical deterioration 20 days after hospitalization, with fever, tachycardia, and mild tachypnea (respiratory rate ranging between 36 and 40 irpm), but with no need for supplementary oxygen therapy. The renal dysfunction was maintained, however, without abnormalities in diuresis and electrolyte/acid-base balance. Chest computed tomography showed bilateral ground-glass opacities diffusely distributed with consolidated areas in the right lung (Figure 1A). At this point, the patient was transferred to a tertiary hospital handling suspected COVID-19 cases. Due to the favorable epidemiological context for SARS-CoV-2 infection and a compatible clinical and radiological condition, the patient received the institutional treatment protocol for SARS-CoV-2 infection (chloroquine 8.5 mg/kg/dose and azithromycin 10 mg/kg/dose, for 5 days). Reverse transcriptase polymerase chain reaction (RT-PCR) for SARS-CoV-2 was positive in two samples.

On the 24th day of hospitalization, the patient started on hemodialysis due to progressive worsening of renal function and reduced urine output, poorly responsive to diuretics. Laboratory tests showed a urea level of 248 mg/dL, creatinine level of 6.59 mg/dL, and serum potassium level of 6.5 mmol/L. The patient's general clinical condition improved, however, renal dysfunction and oligoanuria remained. After the infection was under control, pulse therapy with methylprednisolone (30 mg/kg/dose for 3 days) was started on the 34th day of hospitalization due to the clinical and laboratory suspicion of rapidly progressive glomerulonephritis.

Some laboratory tests used for the etiological clarification of acute kidney injury (AKI) were noteworthy, such as a positive anti-nuclear factor (ANA) test with a fine speckled pattern and reagent nucleus at a titration of 1:160, positive direct coombs test, serum albumin level of 3.3 g/dL, urine 1 microscopic hematuria, a urine protein/creatinine ratio > 2, normal levels of serum complement fractions, a non-reactive perinuclear anti-neutrophil cytoplasmic antibody, negative anticardiolipin IgG and IgM, negative lupus anticoagulant test, non-reactive beta-2-glycoprotein I IgG and IgM, non-reactive anti-Smith, non-reactive anti-native DNA, lactic dehydrogenase level of 497 U/L, transient thrombocytopenia (ranging between 96,000 and 116,000/mm^3^) between days 28 and 31 of her hospital stay. Ultrasound of the kidneys and urinary tract showed normal-sized kidneys with acute nephropathy.

Nineteen days after the diagnostic confirmation of SARS-CoV-2 infection, the patient developed fever (38.3°C), abdominal pain, nausea, vomiting, and moderate respiratory distress (respiratory rate 50 irpm, moderate respiratory effort, and oxygen saturation of 90%). She required oxygen therapy with a concentrating oxygen mask at 5 liters/min. The following findings were highlighted in the clinical decompensation investigation: increased cardiac area on chest X-ray, inflammatory marker changes (ferritin 1,819 ng/ml; D-dimer 10,000 ng/mL), increased cardiac injury markers (peptide natriuretic type B 749 pg/mL; troponin 0.87 ng/mL), and positive blood culture for *Acinetobacter* spp. Echocardiography showed dilation of the left coronary artery trunk (Figure 1B), corroborating the diagnosis of Multisystemic Inflammatory Syndrome in Children (MIS-C) with a “Kawasaki-like” disease.

The patient was immediately treated with a single dose of human immunoglobulin (2 g/kg), acetylsalicylic acid (50 mg/kg/dose for 6 weeks), and broad-spectrum antibiotic therapy due to the bloodstream infection. Five days after starting treatment, the patient had clinical improvement and cardiac injury marker normalization. A coronary artery computed tomography angiography performed 80 days after the immunoglobulin infusion excluded left coronary artery dilation, thus showing cardiac injury recovery and immunoglobulin therapy responsiveness.

**Figure 2:**
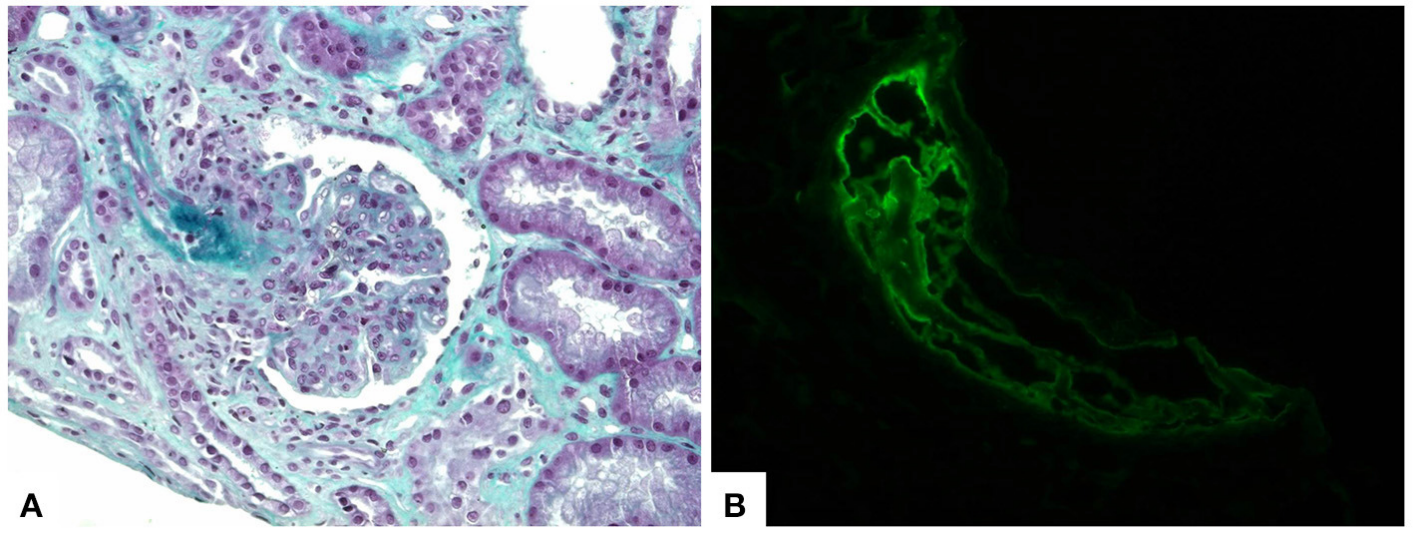
Kidney biopsy showing lupus nephritis and thrombotic microangiopathy findings. **(A)** Histological sections demonstrate proliferative glomerulonephritis and fibrin thrombi in afferent arteriole (trichrome stain, 400×). **(B)** Immunofluorescence staining revealed fibrin deposition within the intravascular thrombi (fibrinogen antiserum, 400×).

However, even with resolution of the infectious context and cardiac decompensation, renal dysfunction and anuria remained, and the patient needed dialysis therapy. A renal biopsy was performed 70 days from admission and revealed 23 glomeruli, all of which were globally sclerotic or in almost global sclerosis, most with fibrous or fibro-cellular growth, and six had capillary tuft necrosis. Some arterioles had concentric wall hyperplasia, while others had endotheliosis. Immunofluorescence showed 12 glomeruli under study conditions with the following findings: IgG ++, IgA +, IgM ++, C3 ++, fibrinogen ++, kappa ++, lambda ++, and C1q ++, which was compatible with “Full-House” Nephropathy, thrombotic microangiopathy and advanced chronic kidney disease (Figures 2A,B). A possible differential diagnosis for the renal condition is class VI Lupus Nephritis, hence, Systemic Lupus Erythematosus (SLE), however this diagnosis may not be stablished since the other criteria may be related to COVID-19 infection.

After clinical improvement of Covid-19 complications and resolution of the investigation of renal disease, the patient was discharged after 125 days of hospitalization for treatment of chronic kidney disease requiring hemodialysis therapy three times a week, and no evidence of renal function recovery.

## Discussion

This report describes the series of clinical complications in a previously healthy child with nephritic syndrome and SARS-CoV-2 infection. These complications included AKI that progressed to chronicity, multisystemic inflammatory syndrome, Kawasaki-like syndrome, and thrombotic microangiopathy.

The input receptor for SARS-CoV-2 in human cells is the angiotensin-converting enzyme 2 (ACE2) receptor. ACE2 expression antagonizes the activation of the classic renin-angiotensin system and protects against organ and system damage (5). Compared to adults, children are believed to have greater ACE2 expression in type II pneumocyte cells, resulting in a lower cytokine release and lower pulmonary capillary permeability, among other immunological mechanisms typical of this age, thus acting as a protective factor against serious COVID-19 complications (6).

The etiology of AKI in SARS-CoV-2 infection is diverse and multifactorial. A recently published review article proposed several mechanisms to justify the renal involvement in COVID-19 patients, including multiple organ dysfunction syndrome, direct renal infection by SARS-CoV-2, AKI after acute respiratory distress syndrome, generalized infection-related mitochondrial insufficiency, and cytokine storm syndrome (2).

The presence of viral particles in renal cells and urine has been reported (1, 3, 4), providing evidence of direct kidney infection in AKI associated with COVID-19. However, an American case series showed no viral inclusions in renal cells or signs of viral particles with immunohistochemistry and *in situ* hybridization in the 17 renal biopsies, including electron microscopy analysis in 13 (7). Thus, the frequency and clinical significance of direct kidney infection in patients with COVID-19 remains unclear. This same series identified a broad spectrum of glomerular and tubular diseases, with podocytopathy being the most common, and five cases of collapsing glomerulopathy (7).

Laboratory tests that reveal renal impairment due to COVID-19 show increased levels of serum creatinine and varying degrees of proteinuria and hematuria (8). A robust prospective cohort performed in a Chinese tertiary hospital reported proteinuria rates of 43.9% and hematuria rates of 26.7% at hospital admission. They also showed an AKI incidence of 6.7%, with a high rate of mortality associated with this complication (9). Urine tests were performed on COVID-19 patients admitted to a medical center in Germany to predict the need for intensive care unit admission, which included findings of SARS-CoV-2 RNA, low anti-thrombin III concentrations, and hematuria (10). This evidence shows that kidney injury recognition and early intervention can limit associated complications and even decrease the risk of long-term chronic kidney disease.

**Figure 3:**
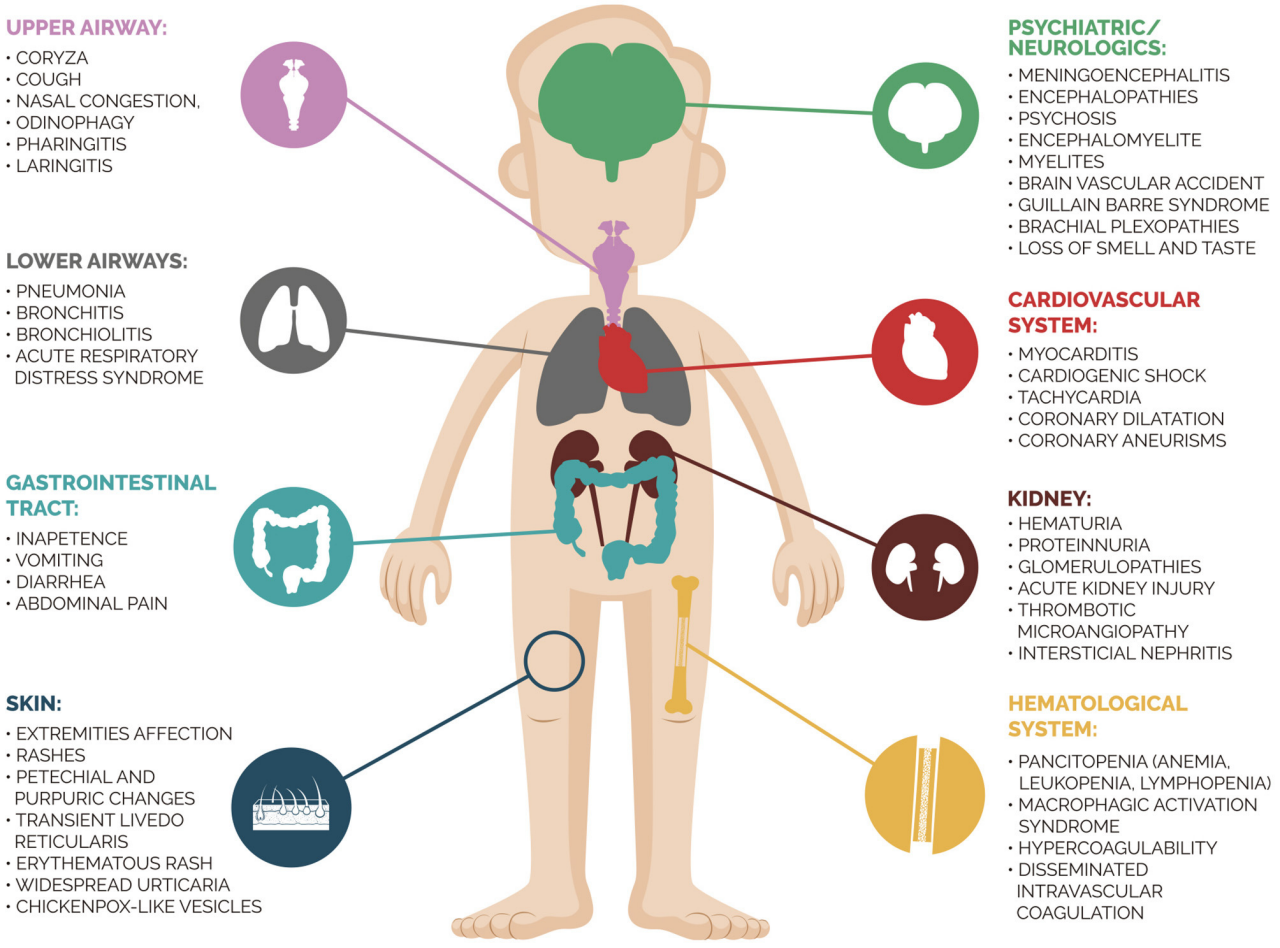
Clinical presentation of Sars-Cov-2 infection in different organs.

Severe SARS-CoV-2 infection in children was rare until mid-April 2020, and respiratory failure seemed to mainly affect patients with comorbidities. However, the European medical community warned the world about the onset of myocarditis, severe inflammatory syndrome, and Kawasaki disease in children with suspected SARS-CoV-2 infection (11). Severe multisystemic inflammatory disease emerging after SARS-CoV-2 infection exemplifies the broad spectrum of possible clinical presentations of this disease. The Centers for Disease Control and Prevention defined the diagnostic criteria of MIS-c as follows: (1) Age <21 years; (2) Fever≥ 38.0°C for ≥ 24 h or report of subjective fever lasting ≥ 24 h; (3) Laboratory evidence of inflammation; (4) Evidence of clinically severe illness requiring hospitalization with multisystem (≥2) organ involvement (cardiac, kidney, respiratory, hematologic, gastrointestinal, dermatologic, or neurological), (5) No alternative plausible diagnoses; (6) Positive for current or recent SARS-CoV-2 infection by RT-PCR, antibody, or antigen test; or exposure to a suspected or confirmed COVID-19 case within the 4 weeks prior to the onset of symptoms. To diagnosis MIS-c, all the criteria must be present and as additional comment, they report that some individuals may fulfill criteria for Kawasaki disease but should be reported if they meet the case definition for MIS-C (12). In our case, the child presented all criteria above, with systemic involvement of heart, gastrointestinal and respiratory system. An interesting study (13) compared clinical and laboratory findings of children and adolescents with MIS-C due to COVID-19 vs. severe acute COVID-19. They observed that MIS-C patients were more likely to be 6 to 12 years old, non-Hispanic black, present cardiorespiratory or cardiovascular involvement, as well as higher C-reactive protein level and lower platelet count. Figure 3 summarizes the multifaceted clinical presentations of SARS-CoV-2 infection. In our case, the patient presented a previous renal dysfunction with a supposed diagnosis of PIGN. Her clinical findings were compatible with nephritic syndrome and no extra-renal abnormality was detected. A new onset of multiorgan involvement after a COVID-19 diagnosis suggested a superimposed disease and the investigation revealed the diagnosis of MIS-C. The patient was prompt treated, allowing her to reach complete recovery of cardiac, gastrointestinal, and respiratory affection.

Although the cause of Kawasaki disease remains uncertain, viral infection seems to trigger genetically predisposed children. Citations in the literature have included the coronavirus family in the pathogenesis of Kawasaki disease (14). Therefore, the development of Kawasaki disease may be the result of a post-viral immune reaction (15). In addition, dramatically improved cardiac function and significantly decreased inflammatory biomarkers after intravenous immunoglobulin reinforces the hypothesis of a post-infectious disease by SARS-CoV-2 (11). A recent Italian cohort reported a strong association between the outbreak of Kawasaki disease and the SARS-CoV-2 epidemic in Bergamo province, showing a monthly incidence that was 30 times higher than that in the last 5 years. Clinically, the disease was more frequent in older children (mean age of 7.5 years) and showed less responsiveness to immunoglobulin therapy (14). In a North American series, Kawasaki-like syndrome was studied in 17 Caucasian patients with SARS-CoV-2 infection, most of whom were previously healthy, with a mean age of 8 years. Initial echocardiographic evaluation showed normal coronary artery dimensions in all patients, although they were described as prominent or echogenic in seven cases. Most of the reported cases showed functional improvement on a follow-up echocardiogram (range 2–18 days after admission). As for laboratory changes, there were high levels of type B natriuretic peptide, ferritin, and D-dimer (16). In a French study, the clinical outcome was favorable in all patients, and moderate coronary artery dilation was detected in 24% of patients during hospitalization (15).

When the patient renal involvement is retrospectively assessed, the hypothesis of PIGN was raised at first, given the presentation in the form of nephritic syndrome in a child with a recent diagnosis of impetigo. However, some issues such as normal complement levels, positive ANA, nephrotic range proteinuria and significant worsening of renal function were remarkable. In the context of PIGN, a polyclonal activation with sequential positivity for autoimmune markers as ANA, anti-DNA and rheumatoid factor has already been described in the literature (17). Furthermore, atypical cases of PIGN with normal complement levels and nephrotic proteinuria has been reported (18, 19). The non-recovering of renal function, two Anti-ANA positive tests, nephrotic range proteinuria and posterior hematologic abnormalities raised the suspicion of a systemic disease that has presented itself with renal injury at first. Another question to be discussed is if renal biopsy findings be a result of a COVID AKI superimposed to PIGN. Histologic abnormalities detected on renal biopsy revealed a glomerular disease associated to thrombotic microangiopathy, with no relevant tubular findings and a previous glomerular disease is present since hospital admission. Despite hematologic findings may be compatible with COVID-19, the previous proteinuria, hematuria, a positive ANA as well as the finding of a “full-house nephropathy” at the renal biopsy raise the suspicion of SLE even with normal serum complement once it may be observed in 25% of patients with focal nephritis and 10% of diffuse nephritis (20, 21). The diagnosis of pediatric SLE is still a matter of debate since it may be performed by different criteria. Our patient fulfills criteria for SLE according to the Systemic Lupus International Collaborating Clinics (SLICC), but not for American College of Rheumatology Criteria (22, 23). The European League Against Rheumatism (EULAR)/ACR criteria (2019) which is still in the process of validation for pediatric patients, states that if there is a more likely alternative explanation for an item than SLE, this item should not be counted. In our case, COVID-19 infection may be associated to hematologic abnormalities than EULAR/ACR criteria may not be applied (24). In this scenario, since there is no agreement among the diagnostic criteria, the diagnosis of SLE may not be stablished at this moment.

A state of hypercoagulability is often found in cases of SARS-CoV-2 infection, which is believed to be secondary to the inflammatory cascade hyperactivation, consequently activating procoagulant pathways. Indicative laboratory findings include increased d-dimer and fibrinogen degradation (4). The activation of the classical complement pathway can be a crucial factor in the development of thrombotic microangiopathy (TMA) in lupus nephritis. In a Chinese study of the lupus population, renal thrombotic microangiopathy had a prevalence of 24.3% (25). This finding is higher to those previously reported, whose prevalence varied between 0.5 and 10% of cases (26). In some studies, lupus nephritis progression in TMA cases was worse than in cases without TMA (26).

There is recent evidence in the literature that TMA pathophysiology in COVID-19 is associated with complement disorders, instead of coagulopathies secondary to sepsis or even disseminated intravascular coagulation, with endothelial dysfunction and microvascular thrombosis being a common denominator for both (27). Therefore, we believe two major factors promoted TMA in the present case, since both lupus nephritis and severe COVID-19 infection have been reported in its occurrence. However, in this report, it was impossible to distinguish which factor was responsible or if both factors showed synergism to trigger the disease. There are three more cases of TMA associated with SARS-CoV-2 infection under genetic investigation for atypical uremic hemolytic syndrome in the same hospital, which will be published in the future.

In summary, we presented a challenging case of a child that presented with an initial clinical picture that resembled an acute post-infectious glomerulonephritis, however, with an atypical progression, once no renal recovery was observed and the patient required renal replacement therapy. In the scenario of a long-term hospital stay, the patient was infected with the novel coronavirus and undergone a multisystemic inflammatory syndrome “Kawasaki-like” with a good cardiac outcome once an early treatment was stablished. A biopsy renal was performed after all infectious and inflammatory complications and was compatible with lupus nephritis, characterizing an autoimmune background, and patients was discharged on hemodialysis. This article highlights the multifaceted clinical presentation of the novel coronavirus infection, SARS-CoV-2, and its potent amplifying effects on preexisting or triggered immunological disorders.

## Data Availability Statement

The original contributions presented in the study are included in the article/supplementary material, further inquiries can be directed to the corresponding author/s.

## Ethics Statement

The studies involving human participants were reviewed and approved by University Hospital, Federal University of Maranhão (HU-UFMA), São Luís, Brazil. Written informed consent to participate in this study was provided by the participants' legal guardian/next of kin. Written informed consent was obtained from the individual(s), and minor(s)' legal guardian/next of kin, for the publication of any potentially identifiable images or data included in this article.

## Author Contributions

PN and GS designed and reviewed the manuscript. JL, LL, TO, and IF took responsibility for the integrity of the data. SO and GS wrote the manuscript and elaborated tables and figures. GS, SO, MM, and PN participated in drafting the article or revising it critically for important intellectual content. NF, DB, LL, KC, and TO are responsible for data collection. All authors read and approved the final version to be published.

## Conflict of Interest

The authors declare that the research was conducted in the absence of any commercial or financial relationships that could be construed as a potential conflict of interest.
